# Supplementary material for: A cytofluorimetric analysis of a Saccharomyces cerevisiae population cultured in a fed-batch bioreactor
Source: PLoS One. 2021 Jun 10;16(6):e0248382. doi: 10.1371/journal.pone.0248382 (PMC8191950; doi:10.1371/journal.pone.0248382)
Supplement: Attachment — Submitted filename: review_2021_1.pdf [file pone.0248382.s001.pdf]

Review of "A cytofluorimetric analysis of a Saccharomyces cerevisiae population cultured in a fed-batch bioreactor"

I have reviewed this manuscript and find it to be a useful contribution to the methodology of bulk culture analysis. In reviewing the paper I downloaded the data using the link provided, and am glad to see that the authors are making that data available.

One concern I had is that the analysis was carried out on a single strain, and the results may differ significantly If this method is applied to a range of industrial *S. cerevisiae* strains. Still, it is made clear in the manuscript that this work is based on a single strain.

The manuscript is well written, and and appropriate for PLOS ONE.

Fred S. Dietrich
